# Sox17 and Other SoxF-Family Proteins Play Key Roles in the Hematopoiesis of Mouse Embryos

**DOI:** 10.3390/cells13221840

**Published:** 2024-11-07

**Authors:** Ikuo Nobuhisa, Gerel Melig, Tetsuya Taga

**Affiliations:** 1 Department of Stem Cell Regulation, Medical Research Institute, Tokyo Medical and Dental University (TMDU), 1-5-45 Yushima, Bunkyo-ku, Tokyo 113-8510, Japan; melig.scr@mri.tmd.ac.jp; 2 Department of Nutritional Sciences, Faculty of Nutritional Sciences, Nakamura Gakuen University, 5-7-1 Befu, Jonan-ku, Fukuoka 814-0198, Japan; 3 Department of Stem Cell Regulation, Medical Research Laboratory, Institute of Science Tokyo, 1-5-45 Yushima, Bunkyo-ku, Tokyo 113-8510, Japan

**Keywords:** hematopoietic stem cells, intra-aortic hematopoietic cell clusters, dorsal aorta, SoxF, Sox17, signaling pathway, fetal liver, bone marrow

## Abstract

During mouse development, hematopoietic cells first form in the extraembryonic tissue yolk sac. Hematopoietic stem cells (HSCs), which retain their ability to differentiate into hematopoietic cells for a long time, form intra-aortic hematopoietic cell clusters (IAHCs) in the dorsal aorta at midgestation. These IAHCs emerge from the hemogenic endothelium, which is the common progenitor of hematopoietic cells and endothelial cells. HSCs expand in the fetal liver, and finally migrate to the bone marrow (BM) during the peripartum period. IAHCs are absent in the dorsal aorta in mice deficient in transcription factors such as Runx-1, GATA2, and c-Myb that are essential for definitive hematopoiesis. In this review, we focus on the transcription factor Sry-related high mobility group (HMG)-box (Sox) F family of proteins that is known to regulate hematopoiesis in the hemogenic endothelium and IAHCs. The SoxF family is composed of Sox7, Sox17, and Sox18, and they all have the HMG box, which has a DNA-binding ability, and a transcriptional activation domain. Here, we describe the functional and phenotypic properties of SoxF family members, with a particular emphasis on Sox17, which is the most involved in hematopoiesis in the fetal stages considering that enhanced expression of Sox17 in hemogenic endothelial cells and IAHCs leads to the production and maintenance of HSCs. We also discuss SoxF-inducing signaling pathways.

## 1. Introduction

Hematopoietic stem cells (HSCs) are a source of hematopoietic cells. HSCs have the ability to self-renew and produce identical HSCs and are also multipotent in their ability to generate all lineages of the hematopoietic system. HSCs can be found in the bone marrow (BM) of adult mice and humans. In mouse ontogeny, primitive hematopoietic cells, hematopoietic progenitor cells of myeloid and erythroid cells, and T- and B-lymphocytes form on embryonic days (E) 7.5~9.5. After this, HSCs form in the aorta–gonad–mesonephros (AGM) region during E10.5 [1,2,3,4] as reviewed in [5,6,7]. HSCs that expand in the fetal liver settle in the BM during the peripartum period. Various transcription factors and signaling molecules are involved in the fetal hematopoiesis of mice [8,9,10] as reviewed in [11,12,13]. In this review, we briefly summarize the hematopoiesis process during mouse ontogeny and describe the function of transcription factors in the Sry-related high-mobility group (HMG) box (Sox) F family, whose downstream genes induce other genes involved in fetal hematopoiesis, with a particular focus on Sox17.

## 2. Hematopoiesis in Mice

HSCs are present in adult BM, and most HSCs remain dormant while in contact with various niche cells such as CXC chemokine ligand 12 (CXCL12)-abundant reticular (CAR) cells, endothelial cells, and so on [14,15]. Demand for hematopoietic cells leads to cell-cycle progression in HSCs via detachment of the dormant HSCs from these niche cells, thereby providing hematopoietic cells [16,17] as reviewed in [18]. To assess the self-renewal capacity and the multipotency of HSCs in vivo, HSC transplantation was performed in lethally irradiated recipients [19,20]. The sustained blood supply from the cells transplanted into recipients for at least 3 months provided evidence that transplanted cells contain long-term reconstituted HSCs (LT-HSCs) [19,20]. Spangrude et al. first reported identifying HSCs in mouse BM by analyzing surface markers [21]. In lineage marker negative (Lin^−^) BM cells expressing the mouse stem cell antigen (Sca-1, a Ly-6A/E molecule) and expressing a low level of T lymphocyte antigen, Thy1.1, the phenotype was enriched in HSCs [22,23]. The *c-Kit* proto-oncogene was highly expressed in BM hematopoietic stem/progenitor cells (HSPCs) [24] and LT-HSCs were found in Lin^−^c-Kit^+^Sca-1^+^ cells [25]. Attempts to enrich BM HSCs using surface markers have continued, and now, LT-HSCs are phenotypically defined as Lin^−^c-Kit^+^Sca-1^+^CD150^+^CD48^−^ cells in adult BM [26]. Lin^−^c-Kit^+^Sca-1^+^ cells in the BM are termed HSPCs. However, HSCs with a low expression level of c-Kit show different features compared with those with a high expression level; c-Kit^low^ HSCs have a myeloid-biased differentiation potential, whereas c-Kit^high^ HSCs exhibit enhanced self-renewal and long-term reconstituted abilities [27]. The administration of interferon (IFN-γ) in mice leads to an increase in the number of Lin^−^c-Kit^+^Sca-1^+^ BM cells and a decrease in the Lin^−^c-Kit^+^Sca-1^+^CD150^+^CD48^−^ BM cells [28]. Moreover, the addition of phorbol ester to human cord blood HSCs leads to an increase in the phenotypic HSC population (CD34^+^CD38^−^CD45RA^−^CD90^+^CD49f^+^) but a reduction in stem cell abilities [29]. These results indicate the limitations of using markers to identify HSCs.

**Figure 1:**
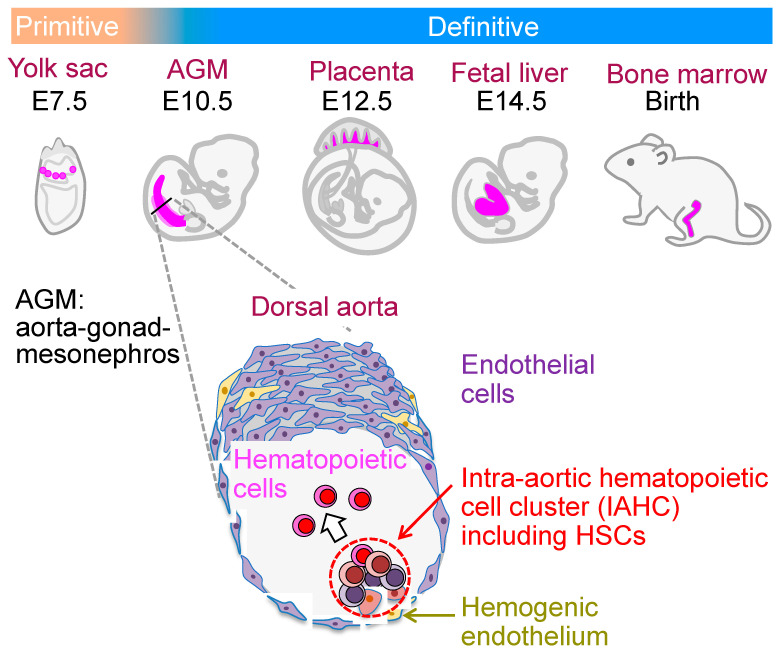
Hematopoietic sites during mouse ontogeny and the emergence of HSCs in the dorsal aorta in the midgestational mouse embryo. Primitive hematopoiesis occurs in the yolk sac at E7.5. Blood islands in the yolk sac are composed of endothelial cells and hematopoietic cells that contain nucleated erythrocytes and macrophages. Definitive hematopoiesis is found in the AGM region, the placenta, and the fetal liver, and finally settle in the BM. In the AGM region at E10.5, HSC-containing IAHCs arise from the hemogenic endothelium.

During development, hematopoietic cells first form in the extra-embryonic yolk sac of a mouse embryo at E7.5 [30] (Figure 1). These hematopoietic cells exist in blood islands surrounded by endothelia, and these hematopoietic cells and endothelia are derived from a common progenitor, hemangioblasts [31]. Hematopoietic cells consist of large nucleated erythrocytes, macrophages, and megakaryocytes, and EMPs form in the yolk sac at around E8.25 [32]. The EMPs form the c-Kit (a marker of adult BM HSPCs)-positive cell clusters in the yolk sac at E9.5 [33]. Adult tissue-resident macrophages originate from these yolk sac EMPs [34]. Lymphocyte progenitors become biased toward B-1 cells (a subtype of B lymphocytes) in the yolk sac and the AGM region at around E9.0 [35]. T cell progenitors are observed in the yolk sac at E9.5 [2].

HSCs are found in the AGM region at E10.5 [36]. Detailed analyses of the AGM regions indicate that the HSC-containing population arises from the hemogenic endothelium, which has the capacity to undergo an endothelial–to–hematopoietic transition (EHT) [37,38,39]. The differentiation process from hemogenic endothelium to hematopoietic cells was observed in a culture of embryonic stem (ES) cells. The FlK-1-expressing mesodermal cells derived from ES cells gave rise to endothelial cells including hemogenic endothelium and vascular smooth muscle cells in an in vitro culture [40,41]. Hematopoietic cells emerged from the hemogenic endothelium in the culture. However, the in vitro expansion of transplantable HSCs was not achieved in the hemogenic endothelium-inducing ES cell culture, except when the expression of *Hoxb4* or *Lhx2* was forced in the mouse ES cells and the expression of seven transcription factors was forced in the hemogenic endothelium derived from human pluripotent stem cells [42,43,44,45]. Recently, transplantable HSCs were generated from human iPSCs [46]. However, further information on the results is awaited. The expansion of HSCs in the AGM region in midgestational mouse embryos was observed in the explant cultures of dissociated AGM cells on a filter with cytokines [47]. Yokomizo et al. first reported on whole–mount immunohistochemistry midgestation to examine protein expression in IAHCs and endothelial cells in the dorsal aorta [39]. Endothelial cell markers, vascular endothelial–cadherin (VEC) and CD31, were expressed in the basal cells in the IAHCs of the dorsal aorta, whereas a hematopoietic cell marker, CD45, was expressed in the apical cells in IAHCs. The c-Kit protein was expressed consistently in all cells of the IAHCs. These data indicated that hematopoietic cluster cells arise from hemogenic endothelia, and the differentiation of hematopoietic cells occurs in IAHCs. The expression of various proteins can be examined in IAHC cells and endothelial cells in the dorsal aorta of mouse embryos via whole-mount immunohistochemistry. Mice deficient in *Runx1*, which is a transcription factor essential for both primitive and definitive hematopoiesis, had no c-Kit^+^ IAHCs in the dorsal aorta, revealing a tight relationship between IAHCs and the emergence of HSCs [39]. 

Transgenic mice expressing Cre recombinase under the control of the *VEC* promoter that is expressed in endothelial cells of the dorsal aorta (*VEC*-*Cre* mice) were used to investigate the effect of target molecules on the production of hematopoietic cells from hemogenic endothelia [37]. The *VEC*-*Cre*-mediated deletion of *Runx1* or *GATA2* resulted in the emergence of little or no c-Kit^+^ IAHCs in the dorsal aorta midgestation [38,48]. Yokomizo et al. have also reported that the leukemia-associated transcription factor hepatic leukemia factor (Hlf) is a marker of HSCs in embryos [49].

The membrane marker proteins of HSCs in adult BM are distinct from those in the AGM region of mouse embryos. VEC^+^ and CD45^+^ cells in the AGM region have the ability to cause long-term reconstitution in vivo [50], whereas CD45^low^c-Kit^high^ cells or CD45^+^c-Kit^+^ cells in the AGM region also include HSCs [51]. Medvinsky et al. have reported that HSCs in IAHCs develop through a multistep process, pro-HSC (CD41^+^CD31^−^CD43^−^CD45^−^ cells) at E9.5, pre-HSC type I (VE-Cad^+^CD41^low^CD43^−^CD45^−^ cells) at E10.5 and E11.5, pre-HSC type II (VE-Cad^+^CD41^low^CD43^high^CD45^+^ cells) at E11.5, and definitive HSCs at E11.5 [52]. The reconstituting ability of cells cultured with OP9 stromal cells for 4 days was observed in their pre-HSCs and pro-HSCs [53]. However, the mechanism by which HSCs mature in IAHCs in contact with endothelial cells in the dorsal aorta remains to be elucidated.

CD34^+^ cells, in which HSCs are enriched, are present in mouse placenta at around E12.5 [54,55,56]. Sodium calcium exchanger *Ncx1*-deficient embryos, which lack heartbeats and circulation, display hematopoietic cells along endothelial cells in the placenta, indicating the emergence of HSCs in the placenta [54]. In the fetal liver at around E12.5, HSCs are greatly expanded and Lin^−^c-Kit^+^Sca-1^+^CD150^+^CD48^−^ LT-HSCs can be detected [57]. Expanded HSCs move from the fetal liver to the BM. Side population (SP) cells identified via Hoechst 33342 staining and flow cytometry are known to contain HSCs and were first reported in adult BM [19]. From the analysis of BM SP cells in the earlier stages, SP cells are not identifiable in 2-week-old mice, but become detectable in 4-week-mice, with their numbers gradually increasing until week 8 [17,58]. Similarly, HSCs in the G0 phase of the cell cycle in adult BM are detected in 4-week-old mice, but not 3-week-old mice [59]. These data indicate that HSCs in the BM start to interact with niche cells at around 4 weeks. Runx1-expressing cells in the yolk sac at E7.5 are marked with the *LacZ* gene using the tamoxifen-inducible system, and the Runx1-expressing cells are detectable among IAHCs at E11.5, HSCs in fetal liver at E14.5, and adult BM [60]. These results suggest that some of the HSCs in BM are derived from Runx1-expressing cells in the yolk sac.

## 3. Phenotypes in Transcription Factors SoxF Family Genes-Deficient Mice

Twenty members of the Sox family can be found in mice and humans [61]. Sox genes can be divided into eight mouse and human subfamilies according to their similarity to the amino acid sequences in the HMG-box, which is a DNA binding domain [61,62]. The HMG-box domain in Sox proteins recognizes the sequence (A/T)(A/T)CAA(A/T)G [63,64]. The subfamily SoxF contains three members, i.e., Sox7, Sox17, and Sox18, that have the HMG-box domain, a β-catenin binding region used to negatively regulate the Wnt signaling pathway, and a transcriptional activation domain [65]. The position of the transcriptional activation domain in the Sox17 protein differs from that in the Sox7 and Sox18 proteins [65].

Heterozygous mice with a deletion of the second exon of the *Sox7* gene exhibit congenital diaphragmatic hernia as adults, while during midgestation, embryos with this deletion show developmental retardation, pericardial edema, and failure in vascular remodeling in the yolk sac [66]. *Sox17*-deficient embryos show defects in tube formation of the gut [67] and are lethal midgestation [68]. A *Sox17* heterozygous mutation leads to an embryonic biliary atresia-like phenotype and hepatitis due to the aberrant cell wall formation in their gallbladders [69,70,71]. A *Sox17* heterozygous mutation in mice results in female subfertility caused by implantation failure [72]. The mesodermal-specific deletion of *Sox17* leads to impaired endocardium differentiation [73]. Apart from mesodermal organs, the transient expression of *Sox17* in pre-myelinating oligodendrocytes, which are of ectodermal origin, is required for the expansion of oligodendrocyte progenitors and differentiation [74,75,76]. Ragged (Ra) mutant mice that have a mutation due to deletion in the transactivation domain in Sox18 exhibit defects in their cardiovascular systems and hair follicle morphology [77]. *Sox18*-deficient embryos on a C57BL6 background display edema and embryonic lethality at around E14.5 [78]. A dominant negative mutation in the *Sox18* gene affects hair follicle development through the silencing of Wnt5a expression [79], and the overexpression of *Sox18* in papillary thyroid carcinoma cells reduces the expression level of active β-catenin [80].

## 4. Function of SoxF Proteins in Early Embryonic and Hematopoietic Development in the Mouse Embryo

Differences have been reported in the expression and function of SoxF family proteins Sox7, Sox17, and Sox18. Sox17 deficiency causes midgestational lethality in mouse embryos alongside growth retardation, posterior patterning defects, and an increase in Annexin V-positive cells in HSCs [67,68], and the knockout of *Sox18* in mice results in the absence of lymphatic vascular or embryonic lethality at E14.5 in a mouse strain-dependent manner [81]. Mice lacking a second exon encoding half of the HMG domain and the transactivation domain in a Sox7 gene die of defects in the cardiovascular system between E11.5 and E14.5 [54]. Sox7 and Sox18 cooperatively act on cardiovascular development, whereas Sox17 functions in the development of arterial vessels [82]. Sox17 is involved in endodermal differentiation and fetal hematopoiesis, while Sox18 is associated with the development of blood and lymphatic vessels [82].

Sox17 was initially known as a marker of endodermal cells [83,84]. Mouse late blastocysts are composed of epiblast, primitive endoderm, and trophectoderm cells. Primitive endoderm and trophectoderm are extra-embryonic tissues included in the yolk sac and placenta. Sox17 expression is detected in the primitive endoderm [85]. The forced expression of Sox17 could not directly induce differentiation from ES cells to the primitive endoderm, whereas a *Sox17*-deficient ES cell-derived primitive endoderm could not differentiate into visceral and parietal endoderm [83]. In ES and iPS cells, a SoxB subfamily member, Sox2, which interacts with Oct4, plays a crucial role in maintaining the stem cell capacity with stemness gene expression. In contrast to the case in ES and iPS cells, the binding partner of Oct4 is changed from Sox2 to Sox17 in endodermal differentiation and the binding sequences of the Sox2/Oct4 complex in the genome are changed to those of the Sox17/Oct4 complex, which induce the expression of the endodermal genes, as revealed by previous chromatin immune precipitation sequencing experiments [86,87]. These results suggest that Sox17 is a regulatory switch for the differentiation of visceral and parietal endoderm. Sox7 is expressed in the primitive endoderm of mouse embryos [84]. Unlike Sox17, the inducible expression of *Sox7* in ES cells shows the differentiation of the primitive endoderm [88]. However, *Sox7*-deficient ES cells differentiate into primitive endoderm normally, indicating that Sox7 has a supplementary role in the differentiation of the primitive endoderm [88].

SoxF proteins are essential for vascular development. Sox7 and Sox18 are expressed in endothelial cells of the blood island in the yolk sac at E7.5 [89] and promote vascular remodeling and angiogenesis in the dorsal aorta and cardinal vein at E8.5 [90]. Heterozygous embryos with deletion of the second exon of the *Sox7* gene midgestation show developmental retardation, pericardial edema, and failure in vascular remodeling in the yolk sac [66]. The Ra mutant mice with a deletion of the transactivation domain in *Sox18* exhibit defects in the cardiovascular system and hair follicle morphology midgestation [77]. An increased expression of Sox17 can be found in the dorsal aorta at E9.5 [89]. Sox7 and Sox18 are widely expressed in arterial and venous vessels, while Sox17 shows specific expression in arterial vessels [91]. Sox18 has an important role in the early stages of lymphangiogenesis from the venous vasculature [78]. A recent study reported that Sox17 promotes endothelial regeneration following endotoxin-induced vascular injury [92]. The molecular mechanism of endothelial cell proliferation involves the hypoxia-induced expression of Sox17, which upregulates cyclin E expression [92].

**Figure 2:**
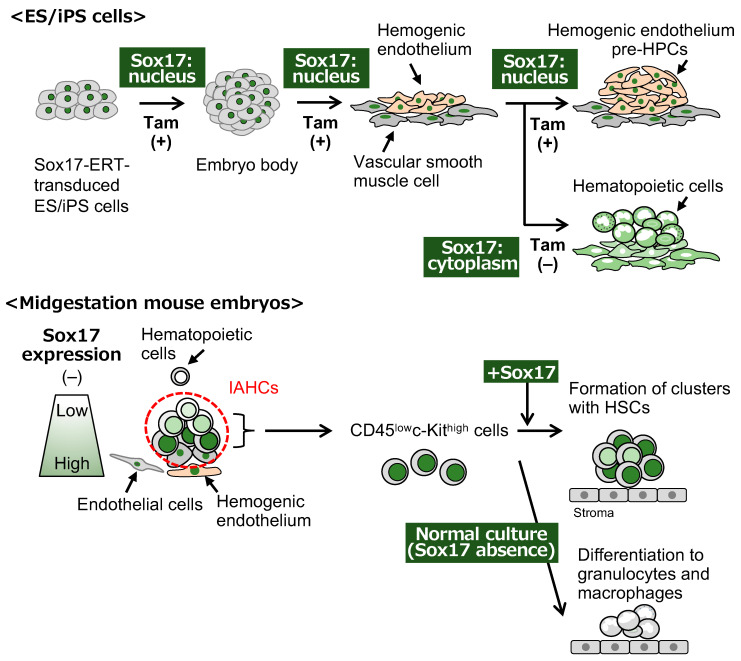
Sox17 has different roles in hemogenic endothelia and IAHCs. Upper panel: *Sox17*-*ERT*-transduced ES cells and iPS cells are subjected to suspended culture with tamoxifen to translocate the Sox17 protein to the nucleus, and embryoid bodies are formed. The *Sox17*-*ERT* transduced cells that express a mesodermal marker in the embryoid bodies are cultured with tamoxifen, and the vascular smooth muscle cells and hemogenic endothelium appear in the culture. The adherent cluster-like structure, in which the hemogenic endothelial cells and pre-hematopoietic progenitor cells (pre-HPCs) proliferate, is observed in the culture with tamoxifen but hematopoietic cells are not able to emerge from the hemogenic endothelium. Withdrawal of tamoxifen to export the Sox17-ERT protein from the nucleus leads to the emergence of hematopoietic cells arising from the hemogenic endothelium. Lower panel: the Sox17 expression level is high in endothelial cells and basal cells in IAHCs of the dorsal aorta, low in apical cells in IAHCs, and none in differentiated hematopoietic cells. Introduction of *Sox17* into CD45^low^c-Kit^high^ cells, which are a component of IAHCs, leads to the maintenance of cell clusters containing HSCs, whereas cultured CD45^low^c-Kit^high^ cells, in which Sox17 expression is gradually lost, differentiate into granulocytes and macrophages. Adapted from references [80,83].

HSCs midgestation express endothelial marker proteins because HSCs in IAHCs arise from hemogenic endothelial cells that are contained in the endothelial cells of the dorsal aorta. Sox17 is highly expressed in Flk-1 (vascular endothelial growth factor receptor 2)-positive cells derived from embryoid bodies at E5.25 [93]. The Flk-1^+^ Sox17^+^ cells have a high capacity to differentiate into hematopoietic cells including T-lymphocytes [93]. In knock-in mice expressing a *GFP* gene under the control of the *Sox17* promoter, LT-HSCs are more concentrated in GFP^+^ Sox17-expressing cells in VEC^+^CD45^+^ LT-HSCs in the E11.5 AGM regions [94]. Sox17 expression is recognized as a hallmark of cells that have the capacity of HSCs. Tamoxifen-induced Sox17 nuclear translocation in *Sox17*-*ERT*-transduced ES cells expands adherent cell aggregates, which are mainly composed of CD34^+^CD43^+^CD45^-/low^VEC^+^ hemogenic endothelial cells, on stromal cells because Sox17 inhibits the differentiation of hematopoietic cells from hemogenic endothelial cells into their aggregates [95]. The removal of tamoxifen from the culture of *Sox17*-*ERT*-transduced hemogenic endothelium-like cells results in the emergence of hematopoietic cells [95] (Figure 2). The expression level of Runx1 detected in IAHCs along the dorsal aorta in the AGM region is higher than that in the endothelial cells. The level of Sox17 expression is lower in IAHCs than in endothelial cells [96,97]. A detailed analysis of in situ hybridization reveals that the *Sox7* and *Sox17* genes are expressed in basal cells in IAHCs and endothelial cells of the dorsal aorta midgestation [98] (Figure 2). Whole-mount immunohistochemistry also showed Sox17 expression in basal cells, at least in part, at the protein level [96,97]. The forced expression of *Sox17* in CD45^low^c-Kit^high^ cells, which are one component of IAHCs in the E10.5 AGM region, maintains the formation of cell clusters with hematopoietic activity in co-culture with OP9 stromal cells in the presence of stem cell factor (SCF), interleukin(IL)-3, and thrombopoietin (TPO) [98] (Figure 2). A shutdown of Sox17 expression in Sox17-transduced cells results in an induction of hematopoietic differentiation in cell clusters [98]. Sox17-transduced cells show a long-term repopulating ability in vivo when intra-BM transplantation occurs in lethally irradiated mice [98]. These results indicate that Sox17 plays a key role in the emergence and self-renewal of HSCs in IAHCs.

Sox7 expression is observed in the future blood island area of the yolk sac at E7.5 and the endothelial cells in the dorsal aorta of embryos at E9.5 and E10.5 [89,99]. The basal cells in IAHCs found alongside endothelial cells at E10.5 express Sox7 [97]. In the differentiation culture system of ES cells, hemogenic endothelia, not hematopoietic progenitors, express the *Sox7* gene [89,100]. The inducible expression of *Sox7* in ES cells reveals the expression of endothelial markers, not hematopoietic markers [100]. The forced expression of *Sox7* inhibits hematopoietic differentiation in the ES culture system [89,100]. Considering the expression pattern and the function of Sox7, Sox7 is involved in the generation of hemogenic endothelia and the negative regulation of hematopoietic cell production. The overexpression of *Sox7* in CD45^low^c-Kit^high^ AGM cells maintains cell clusters with a hematopoietic capacity [98]. These data show the functional redundancy of the SoxF family members in the culture of cluster cells.

The inducible expression of the *Sox18* gene in ES cells leads to the production of hematopoietic cells and the expression of the *GATA1* and *Scl* genes, which are important for hematopoiesis, whereas inducible Sox18 expression inhibits the maturation of hematopoietic cells in yolk sac hematopoiesis [101]. *Sox18* is expressed in all hematopoietic cluster cells and endothelial cells in the dorsal aorta [98]. The forced expression of *Sox18* in the cluster cells of E10.5 AGM regions leads to the formation of clusters with a hematopoietic capacity [98]. However, this clustering induced by *Sox18* is transient and cannot be maintained in the long term [98]. Sox18 is likely to possess different properties during fetal hematopoiesis.

The results of the EHT in hemogenic endothelia derived from human ES cells have been reported. Sox17 expression is used as a marker of hemogenic endothelia in human ES cells [95,102]. Sox17 induces the expression of the *HoxA* gene in hemogenic endothelia derived from human pluripotent stem cells, and the determination of arterial cell fate for Sox17-expressing hemogenic endothelial cells is observed with the activation of Notch signaling [103]. Arterial hemogenic endothelia eventually lead to the production of lympho-myeloid hematopoietic progenitor cells (HPCs) [103].

## 5. Relationship Between SoxF Proteins and Signal Molecules in Midgestation Hematopoiesis in Mice

**Figure 3:**
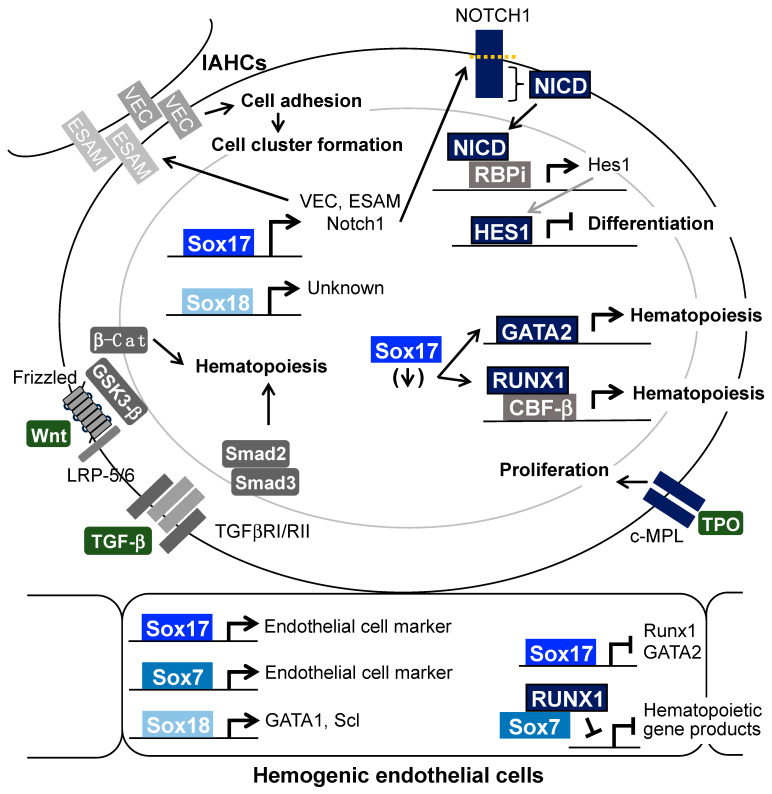
Contribution of Sox17-induced gene products to the maintenance of the hematopoietic capacity and cluster formation. In the hemogenic endothelial cells (the lower part of the figure), Sox7 and Sox17 activate the genes encoding endothelial cell marker proteins and Sox18 induces expression of the *GATA1* and *Scl* genes. Moreover, Sox7 inhibits the interaction of Runx1 with its cognate DNA sequence by direct binding of Sox7 to Runx1. In the hemogenic endothelial cells, Sox17 decreases the expression of *Runx1* and *GATA1*, which are essential transcription factors for definitive hematopoiesis, by binding to the *Runx1* and *GATA2* promoter regions and inhibiting transcription. In the IAHCs (the upper part of the figure), Sox17 directly activates the gene expression of *Notch1*. Notch intra-cellular domain (NICD), which is an activated form of Notch1, induces the expression of *Hes1*, and Notch1- and Hes1-induced gene products are involved in the inhibition of differentiation. Adherent molecules VEC and ESAM, which are directly induced by Sox17, have roles in forming cell clusters with hematopoietic capacity. The reduced expression of Sox17 in IAHCs leads to the increased expression of Runx1 and GATA2. The TPO/c-Mpl signaling pathway is required for the proliferation of Sox17-transduced cells. The involvement of the Wnt signaling pathway and the TGF-β signaling pathway in maintaining the hematopoietic capacity of IAHCs by Sox17 is reported. Sox18 target genes in IAHCs have not been identified.

SoxF transcription factors induce the expression of various genes involved in hematopoiesis and cluster formation in the fetal stage. In this section, we focus on the molecular mechanism behind how SoxF proteins functionally interact among each other and with other signaling molecules (Figure 3).

### 5.1. Notch

The Notch signaling pathway plays significant roles in fetal hematopoiesis. In a RNA in situ hybridization analysis, the genes for *Notch1* and *Notch4*, and their ligands—*Jagged1*, *Jagged2*, and *Delta-like4*—were found to be expressed in IAHCs and endothelial cells of the dorsal aorta midgestation [10]. The expression of Notch1 at the protein level was also confirmed in IAHCs and endothelial cells [95,96]. The stimulation of CD31^+^ endothelial cells in the E11.5 AGM region with a Notch ligand Jagged1 led to increased activation of the Notch1-expressing hemogenic endothelium [104]. Moreover, AGM cells in co-culture with stromal cells expressing another Notch ligand, delta-like 1, showed an increased capacity to form colonies in semisolid medium and an increased ability to repopulate hematopoietic cells in vivo [97,105]. Sox17 directly interacts with two Sox-binding consensus sequences in the *Notch1* promoter and induces *Notch1* gene activation [94,97]. Hes1, a downstream factor of the Notch signaling pathway, was found to be expressed in IAHCs and endothelial cells of the dorsal aorta by in situ hybridization, whereas gene expression of *Hes5*, another downstream factor of the Notch signaling, was not observed in these cells [106]. Hes1 has the ability to protect HSCs from various stresses in embryos [107]. *Hes1* and *Hes5* double-knockout embryos midgestation lose their hematopoietic capacity [106]. *Sox17*-transduction in cells derived from IAHCs of the E10.5 AGM regions increases the expression of *Hes1* and *Hes5* genes [97]. The knockdown of *Notch1* and *Hes1* expression in the *Sox17*-transduced cells leads to a decrease in their hematopoietic capacity [97]. These results indicate that the Sox17—Notch1—Hes1 pathway is important for AGM hematopoiesis. The ability of HSCs in the fetal liver and BM to reconstitute cells is dependent on the level of Notch activation [108], whereas *VEC*-*Cre*-driven *NICD*-expressing embryos exhibit a decreased number of hematopoietic cells [109]. These data suggest that a certain level of Notch signaling strength is required for the emergence of hematopoietic cells from endothelial cells. As for other SoxF family members, Sox7 induces *Jagged1*-expression and activates the Notch1 signaling pathway in human kidney endothelial cells [110] and Sox18 induces *Notch1* expression in smooth muscle cells [111].

### 5.2. Runx1 and GATA2

Compared with endothelial cells, the expression levels of Runx1 and GATA2 are increased in IAHC cells via the EHT, whereas the expression levels of *Sox7* and *Sox17* are decreased in IAHC cells [96]. A reason for the decreased expression of *Runx1* and *GATA2* in endothelial cells is that Sox17 directly interacts with the *Runx1* and *GATA2* promoters and inhibits their expression [96]. The complex formed between Sox7 and Runx1 inhibits induction of the Runx1-dependent gene [99]. A decrease in *Sox7* expression induces the interaction of Runx1 with CBF-β, followed by activation of the Runx1 complex-inducible gene for fetal hematopoiesis [99,112]. In the endothelial cells of the dorsal aorta midgestation, Sox17, which directly binds to the *Runx1* and *GATA2* promoters, represses the expression of *Runx1* and *GATA2*, resulting in the absence of genes involved in hematopoiesis [96] (Figure 3).

### 5.3. Adherent Molecules

Sox7 has a role in inducing *VEC* expression and mediating cell adhesion as a result of the interaction of Sox7 with the Sox binding consensus sequences in the promoter of the *VEC* gene [100]. Sox17 also binds to the promoter region of the *VEC* gene and the gene for another adherent molecule, the *endothelial cell-selective adhesion molecule* (*ESAM*) gene [113]. ESAM is a marker of HSCs [114,115] and has an important role in hematopoiesis in fetal liver [116]. The treatment of *Sox17*-transduced cluster cells with short-hairpin RNAs against *VEC* and *ESAM* genes decreased the formation of cluster cells with hematopoietic capacities in a culture with stromal cells [113]. These results indicated the positive effect of SoxF, at least Sox17, on cluster formation and the release of hematopoietic cells from clusters via the reduced expression of cell adherent molecules.

### 5.4. The Wnt Signaling Pathway

Several studies have reported correlations between SoxF proteins and the Wnt signaling pathway in cancer cells and mesodermal cells [82,117]. It is known that the Wnt signaling pathway positively regulates stem cell activity. Recombinant Wnt3a protein acts to enhance the self-renewal capacity of HSCs in adult BM in vitro [118] and to increase hematopoietic progenitors in E8.5 yolk sac [33]. Overexpression of the canonical Wnt signaling target, β-catenin, in the HSCs of mouse BM induces an expansion of HSCs [119]. Moreover, inhibition of the Wnt signaling pathway by a soluble form of the frizzled cysteine-rich domain that inhibits the interaction of Wnt protein with the frizzled receptor, and Axin, which induces the degradation of β-catenin, prevents the growth of adult BM HSCs in vitro and causes a reduction in the reconstitution capacity of Axin-transduced HSCs when transplanted into mice [119]. Transient treatment with a GSK3-β inhibitor (an activator of β-catenin) induces the differentiation of hemogenic endothelial progenitors from human iPS cells [120] and a Wnt agonist supports the differentiation of hemogenic endothelium and hematopoietic cells from human pluripotent stem cells [121,122]. In AGM hematopoiesis, the accumulation of β-catenin is observed in the nuclei of endothelial cells attached to IAHCs in the dorsal aorta [123]. Treatment of explant cultures of embryos midgestation with the GSK3-β inhibitor increases their hematopoietic capacity in vitro and in vivo [123]. However, the involvement of the Wnt signaling pathway in the formation and proliferation of IAHCs remains to be elucidated.

### 5.5. The TGF-β Signaling Pathway

The TGF-β signaling pathway negatively regulates the transition from hemogenic endothelia to hematopoietic cells. TGF-β negatively regulates the generation of hematopoietic cells from ES cells in in vitro culture systems [124] and an inhibitor of TGF-β signaling induces the generation of hematopoietic cells from hemogenic endothelia derived from ES cells [125]. The antagonist of activin, a member of the TGF-β family, induces the differentiation of hemogenic endothelium and hematopoietic cells from human pluripotent stem cells. Moreover, TGF-β receptor *Acvrl1* heterozygous mutation in mouse ES cells leads to increased production of hematopoietic cells [126]. The receptors of TGF-β and activated forms of Smad2/3, which are positive regulators of the TGF-β signaling pathway, are expressed in endothelial cells of the dorsal aorta, but not IAHCs, in midgestational mouse embryos [126]. Activation of the TGF-β signaling pathway shows induction of the Sox17 expression in respiratory epithelia [127] and differentiated cultures of ES cells to generate hematopoietic cells [124]. Runx1 expression induced in E13.5 endothelial cells via stimulation of the TGF-β signaling pathway leads to increased efficiency in the specification of hemogenic endothelial cells [128]. A recent study reported that histone deacetylase (HDAC) 1 and 2 promote EHT by reducing the expression of the negative regulators in the TGF-β signal pathway, Smad6, Smad7, and so on [129]. However, whether the TGF-β signaling pathway is involved in the maintenance of the hematopoietic capacity in IAHCs remains unclear.

### 5.6. Thrombopoietin/c-Mpl

TPO is a positive regulator of HSC activity in adult BM [130]. The in vitro expansion of HSCs in serum/albumin-free culture is largely driven by the addition of TPO [131]. The expression of TPO and their TPO receptor c-Mpl is observed in the AGM region and the fetal liver at E10.5 and E11.5 [132]. As observed in an in vitro culture of Sox17-transduced cells derived from the AGM region with stromal cells, TPO is required for the formation of cell clusters with a hematopoietic capacity [133]. c-Mpl is expressed in IAHC cells in the E10.5 dorsal aorta, and Sox17-transduced cells also maintain the expression of c-Mpl [133]. These data suggest the involvement of Sox17 in maintaining hematopoietic capacity via activation of the TPO/c-Mpl signaling pathway.

## 6. Role of SoxF Family Members in Hematopoiesis of the Fetal Liver and Adult BM

HSCs are expanded in the fetal liver [5]. The expression and function of Sox18 have not been reported in studies of HSPCs in the fetal liver and BM. We mainly focus on studies of Sox17 and Sox7 in hematopoiesis in the fetal liver and BM.

The level of *Sox17* expression is high in the fetal liver compared with that in the newborn liver and 2 week BM [134]. *Tie2*-*Cre* mice express Cre recombinase in the endothelial cells and HSPCs of embryos and *Tie2*-*Cre*-driven *Sox17*-conditional knockout embryos die at E12.5 due to a lack of hematopoiesis in the yolk sac and fetal liver [135]. *Sox17*-conditional knockout is achieved by administrating pIpC to *Mx-1*-*Cre*:*Sox17^loxP/loxP^* mice [68]. The administration of pIpC on days 2, 4, and 6 after birth leads to a decrease in the absolute number of HSCrs in the BM and spleen and death before day14, whereas administration 6 weeks after birth does not cause lethality or any significant decrease in the number of HSCs [68].

The ectopic expression of *Sox17* in adult HSCs positively affects their ability to retain their self-renewal capacity and their multipotency after transplantation [134]. Moreover, the introduction of Sox17 promotes the expression of endothelial cell markers; *VEC*; and a macrophage marker, *Mac-1*, which are markers of fetal HSCs, in adult BM HSCs [134,136]. Sox17 might directly induce the expression of fetal marker genes, e.g., *VEC*, in *Sox17*-transduced HSCs. The transplantation of *Sox17*-transduced HSCs derived from the adult BM causes a markedly reduced chimerism of lymphocytes compared with those of other lineages [134]. *Sox17*-transduced cells derived from IAHCs in the AGM region have a decreased capacity to differentiate into lymphocytes, especially B-lymphocytes in vivo [98]. The forced expression of *Sox7* in adult HSCs also leads to reduced differentiation of B-lymphocytes, not T-lymphocytes after transplantation [137]. However, the mechanism by which lymphocytes are inhibited in *Sox17*-transduced cells remains to be understood.

*Sox17*-transduced HSC-transplanted mice had a shorter lifespan than control mice [134]. Moreover, the secondary transplantation of *Sox17*-transduced BM cells 4 months after transplantation resulted in shorter survival of the transplanted mice [134]. Sox17 is an important factor in identifying the features of fetal HSCs, and the ectopic expression of *Sox17* in the HSCs of BM is sufficient for maintaining stem cell capacity. Moreover, the sustained expression of *Sox17* in HSCs for a long time leads to leukemogenesis. After transplantation of *Sox17*-transduced cells from IAHCs, the absolute number of common myeloid progenitors increased abnormally [138]. *Sox17*-transduced common myeloid progenitors and granulocyte/macrophage progenitors, not vehicle-transduced common myeloid progenitors and granulocyte/macrophage progenitors, were maintained in the culture with stromal cells [138]. Mice transplanted with *Sox7*-transduced cells showed increased spleen and liver size, in which disruption of the spleen structure was also found [137]. These results suggest that the sustained expression of *Sox7* and *Sox17* in HSCs leads to leukemogenesis.

## 7. Conclusions

In this review, we have focused on hematopoiesis in fetal mice regulated by the SoxF family of transcription factors. Particular attention has been paid to Sox17, which induces the expression of adherent molecules, resulting in the formation of hematopoietic clusters, and regulates the expression of genes for signaling molecules involved in the maintenance of the hematopoietic capacity of HSPCs in the dorsal aorta midgestation.

## Data Availability

Not applicable.
